# Antiretroviral Therapy to Prevent HIV Acquisition in Serodiscordant Couples in a Hyperendemic Community in Rural South Africa

**DOI:** 10.1093/cid/ciw335

**Published:** 2016-05-20

**Authors:** Catherine E. Oldenburg, Till Bärnighausen, Frank Tanser, Collins C. Iwuji, Victor De Gruttola, George R. Seage, Matthew J. Mimiaga, Kenneth H. Mayer, Deenan Pillay, Guy Harling

**Affiliations:** 1 Department of Epidemiology; 2 Department of Global Health and Population; 3 Department of Biostatistics, Harvard T. H. Chan School of Public Health; 4 Department of The Fenway Institute, Fenway Community Health; 5 Department of Medicine, Beth Israel Deaconess Medical Center, Boston, Massachusetts; 6 Departments of Behavioral and Social Sciences and Epidemiology, Institute for Community Health Promotion, Brown University School of Public Health, Providence, Rhode Island; 7 Africa Centre for Population Health, Mtubatuba; 8 Faculty of Health Sciences, University of KwaZulu-Natal, Durban, South Africa; 9 Division of Infection & Immunity, University College London, United Kingdom

**Keywords:** HIV, treatment as prevention, serodiscordant couples, antiretroviral therapy, South Africa

## Abstract

***Background.*** Antiretroviral therapy (ART) was highly efficacious in preventing human immunodeficiency virus (HIV) transmission in stable serodiscordant couples in the HPTN-052 study, a resource-intensive randomized controlled trial with near-perfect ART adherence and mutual HIV status disclosure among all participating couples. However, minimal evidence exists of the effectiveness of ART in preventing HIV acquisition in stable serodiscordant couples in “real-life” population-based settings in hyperendemic communities of sub-Saharan Africa, where health systems are typically resource-poor and overburdened, adherence to ART is often low, and partners commonly do not disclose their HIV status to each other.

***Methods.*** Data arose from a population-based open cohort in KwaZulu-Natal, South Africa. A total of 17 016 HIV-uninfected individuals present between January 2005 and December 2013 were included. Interval-censored time-updated proportional hazards regression was used to assess how the ART status affected HIV transmission risk in stable serodiscordant relationships.

***Results.*** We observed 1619 HIV seroconversions in 17 016 individuals, over 60 349 person-years follow-up time. During the follow-up period, 1846 individuals had an HIV-uninfected and 196 had an HIV-infected stable partner HIV incidence was 3.8/100 person-years (PY) among individuals with an HIV-infected partner (95% confidence interval [CI], 2.3–5.6), 1.4/100 PY (.4–3.5) among those with HIV-infected partners receiving ART, and 5.6/100 PY (3.5–8.4) among those with HIV-infected partners not receiving ART. Use of ART was associated with a 77% decrease in HIV acquisition risk among serodiscordant couples (adjusted hazard ratio, 0.23; 95% CI, .07–.80).

***Conclusions.*** ART initiation was associated with a very large reduction in HIV acquisition in serodiscordant couples in rural KwaZulu-Natal. However, this “real-life” effect was substantially lower than the effect observed in the HPTN-052 trial. To eliminate HIV transmission in serodiscordant couples, additional prevention interventions are probably needed.

## Abstract (precis)

We assessed the role of antiretroviral therapy (ART) on human immunodeficiency virus (HIV) acquisition in serodiscordant couples in KwaZulu-Natal, South Africa. ART use was associated with a 77% reduction in HIV acquisition risk, suggesting ART is highly effective for prevention in population-based settings.

Human immunodeficiency virus (HIV) transmission within stable heterosexual partnerships is thought to be a major contributor to new HIV infection in sub-Saharan Africa (SSA) [1–3]. In the era before antiretroviral therapy (ART), HIV incidence was estimated at approximately 5 cases per 100 person-years (PY) among men and 10/100 PY among women in serodiscordant relationships in Tanzania [4] and Uganda [2]. Higher HIV incidence among women in serodiscordant relationships may be related to higher per-act probability of HIV transmission among women than among men [5]. In some studies from SSA, marriage has been implicated as a risk factor for HIV acquisition among women [6, 7], perhaps owing to greater vulnerability to HIV within marriage (eg, due to difficulty negotiating condom use) [8–11].

The introduction of ART has resulted in dramatic decreases in HIV transmission globally [12–16]. The landmark HPTN-052 study demonstrated that immediate ART initiation can nearly eliminate ART transmission in serodiscordant couples [12]. A recent meta-analysis showed greatly reduced HIV transmission in serodiscordant couples in whom the HIV-infected partner is virally suppressed [13]. Increasing ART coverage at the community [14] and household [16] level is associated with decreased HIV transmission, and modeling studies have suggested that HIV transmission could be eliminated at 90% ART coverage [17]. However, estimates of the ART effect in preventing HIV transmission has primarily arisen from clinical randomized trials and clinical cohort studies. Participants in these studies were enrolled through either serodiscordant couple clinics or partner testing programs. These studies were highly controlled and resource-intensive and thus do not represent the “real-life” settings of resource-poor public-sector health systems in the hyperendemic communities of SSA. For instance, in HPTN-052 viral suppression was >89% among individuals immediately initiating ART [18]; in contrast, in public-sector ART clinics in KwaZulu-Natal viral load suppression was 77% [19]. Such differences in viral load suppression are likely to lead to differential effectiveness of ART in preventing HIV transmission. Importantly, all individuals in the prior studies were aware of their HIV status and had disclosed their status to their partners [20–25]. In “real-life” settings in SSA, individuals commonly do not disclose their HIV status to their partners for fear of negative consequences to their lives [26].

To date, no empirical estimates exist of the effect of ART on HIV transmission in “real-life” populations of serodiscordant couples and in settings with resource-poor health systems. In this study, we aim to establish such evidence in an HIV hyperendemic rural community in KwaZulu-Natal.

**Figure 1.**
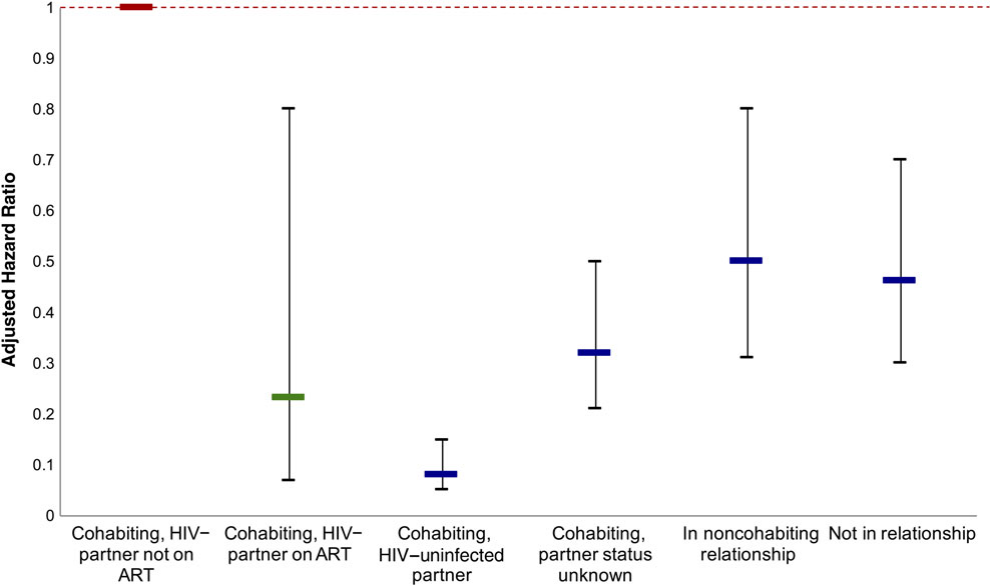
Adjusted hazard ratio for each partnership category compared with cohabiting couples with human immunodeficiency virus (HIV)–infected partners not receiving antiretroviral therapy (ART) (referent category). The interval-censored time-updated proportional hazards model was adjusted for age, sex, educational attainment, household wealth, concurrent partners, and condom use. The interrupted line indicates the referent category.

## METHODS

### Study Setting

Data arose from the population-based longitudinal surveillance program conducted by the Africa Centre for Population Health (hereafter, “Africa Centre”) [27]. This surveillance program is located in a predominantly rural community of uMkhanyakude district in the province of KwaZulu-Natal. The Africa Centre has conducted an HIV surveillance program since 2003, including confidential HIV testing, collection of sexual history and behaviors, relationship status, and household demographic data. The surveillance includes all members of all households located in the 438-km^2^ demographic surveillance area.

KwaZulu-Natal is the province that bears the largest HIV burden in South Africa [28–30]. Age-specific incidence peaks at >6/100 PY among women in KwaZulu-Natal and 4/100 PY among men [14, 31, 32]. Adult HIV prevalence in this region is 29% [33], and only 34% of working-age adults are employed [34]. ART coverage has increased rapidly since 2004, primarily via nurse-led public sector ART programs [14, 33], but rates of viral suppression and immunologic recovery among ART patients are comparatively low [19]. ART is provided primarily in primary care clinics run by nurses [19]. Although ART coverage has expanded rapidly [14], the program suffers from more drug stockouts, and greater difficulty with retention in care, leading to lower viral suppression compared to HPTN-052 [18, 19].

### Participants and Procedures

Data were available from January 2005 through December 2013. Participants were included in this analysis if they had a first negative HIV test result followed by ≥1 subsequent HIV test (regardless of result) and, for those in stable relationships, reported only a single stable partner. Baseline was considered the earliest date at which an individual tested HIV negative.

Ethical approval for data collection, linkage, and analysis was obtained from the University of KwaZulu-Natal Biomedical Research Ethics Committee, and written informed consent was obtained from all participants. The analyses presented here used only secondary and anonymized data and were thus exempted from additional ethical review by the Institutional Review Board.

### Outcome Ascertainment

The primary outcome for analyses was the time to HIV seroconversion, where seroconversion required ≥1 negative HIV test results followed by ≥1 positive result.

### Exposure Ascertainment

The primary exposures of interest were the HIV and ART status of each respondent's partner. In this region, a large number of individuals are in primary, cohabiting, “conjugal relationships” (henceforth, “stable partnerships”), in which they are members of the same household, may have had children together, and are socially recognized as partners [35]. These partnerships are more common among older than among younger individuals, who more frequently live in households headed by their parents or kin. Stable partnerships were identified via demographic household surveillance data. Unique Africa Centre identification codes were used to link partners to one another. Stable partnerships remain under observation until they are reported to have ended by either partner during a household visit.

The HIV status of each stable partner was ascertained from HIV surveillance data. Respondents were considered to be in a serodiscordant relationship if their stable partner had tested positive before or at the same time as the respondent's current negative HIV test, or if the partner had initiated ART before the respondent's current test. Respondents were considered to be in a seroconcordant HIV negative relationship if their partner tested HIV negative on or after the day the respondent him or herself tested negative. Respondents were censored at the end of the relationship, on the date of their first positive HIV test result, or on the date of their last negative HIV test result, whichever was first.

To determine the ART status of HIV-infected partners, we linked demographic surveillance data to information from the Hlabisa HIV Treatment and Care Programme, which provides ART through 17 public-sector primary care clinics in the area which includes the Africa Centre surveillance area. HIV-infected partners were defined as receiving ART at the time of a demographic surveillance visit if they had a record of having initiated ART before that date.

Respondents not in stable partnerships were categorized as being in another type of relationship or not, based on self-reported relationship status. Participants who reported being married or engaged, but not, cohabiting with their partner, were coded as being in a noncohabiting relationship. Those who did not report a current relationship were coded as such. Partnership, HIV, and ART status could vary over time and we thus treated them as time-varying variables in our analyses. Individuals could contribute person-time to multiple exposure categories.

### Covariates

Time-varying potential confounders included respondent-reported current completed education (none or primary, 0–7 years; secondary, 8–12 years; tertiary, >12 years), household wealth (quintiles of the first component identified by principal components analysis of 32 household assets and characteristics), having had >1 partner in the past 12 months, and inconsistent condom use with any partner in the past 12 months. Additional covariates included age at baseline and sex.

### Statistical Methods

Exact HIV seroconversion dates are not typically observed in cohort study research. Instead, the event time is “interval censored,” in that it is known that it occurs within an interval of time (between the dates of the last negative and the first positive HIV test result). The standard approach to the use of interval-censored data is to impute an exact seroconversion date, for example, by assuming that the HIV seroconversion occurred at the midpoint between the dates of the last negative and the first positive HIV test [36]. However, this approach has been demonstrated to lead to underestimated standard errors and can lead to misclassification if the date of a time-varying exposure is known with precision but not the event date of HIV seroconversion [36]. Here, the date of exposure is known with precision because ART initiation dates are extracted from clinical HIV treatment program data, while exact HIV seroconversion dates are not known with precision owing to the annual nature of the HIV surveillance. To avoid the problems associated with exact seroconversion date imputation, we fitted interval-censored parametric proportional hazards models, which appropriately account for both uncertainty regarding HIV seroconversion timing and the time-varying nature of the exposure and potential confounders [37, 38].

A series of 3 models were included for each contrast of interest (HIV-infected vs HIV-uninfected partner; HIV-infected partner receiving vs not receiving ART). Model 1 contained an indicator term for each partnership status category, the individual's age, sex, and an indicator for year of observation. In model 2 we added educational attainment and household wealth as additional covariates. In model 3, we further added sexual behavior variables. For models considering the effect of partner HIV status on HIV incidence, the referent category was having an HIV-uninfected partner. For models considering the effect of partner ART status, the HIV-infected partner's ART status was modeled as the proportion of the interval that was covered by ART. This indicator variable could thus range from 0 to 1.

Several sensitivity analyses were performed. First, given that ART use does not result in immediate viral suppression, we modeled the partner's ART use with a “wash-in” period of 30 days, assuming that the first 30 days after ART initiation were untreated in an interval-censored model. Second, we repeated our analyses with a Cox proportional hazards model using a midpoint imputed HIV seroconversion date, to compare the results based on the interval-censored approach to those based on the more standard midpoint imputation approach. Analyses were performed using SAS 9.2 (SAS Institute Inc, Cary, North Carolina) and Stata 13.1 software (StataCorp, College Station, Texas).

## RESULTS

Between January 2005 and December 2013, 17 016 individuals contributing 60 349 PY of follow up met the inclusion criteria. Of 2029 individuals with a cohabiting partner during the follow-up period, 196 individuals had an HIV-infected cohabiting partner, and 1846 individuals had an HIV-uninfected stable partner. Of individuals with an HIV-infected cohabiting partner, 20 were receiving ART at baseline, and a further 56 started ART during the follow-up period. Individuals were tested for HIV between 2 and 9 times (24.4% were tested twice, 21.4% 3 times, 32.1% 4–5 times, 17.5% 6–7 times, and 4.6% 8 or 9 times). The median time between tests was 374 days (interquartile range, 352–700 days). Table 1 lists baseline characteristics for the study sample. Approximately 63% of the study sample was female, and women tended to be older than men (mean age, 36 vs 29 years, respectively). At baseline, 29% of participants reported being aware of their HIV status.

**Table 1.** Baseline Descriptive Characteristics by Respondent Sex

Characteristic	Respondents, No. (%)^a^		
	All (N = 17 016)	Male (n = 6355)	Female (n = 10 661)
Age at baseline, mean (SD), y	33.2 (20.5)	28.6 (17.9)	36.0 (20.5)
Educational attainment, %			
None or primary (0–7 y)	40.8	36.8	43.2
Secondary (8–12 y)	55.3	59.0	53.0
Tertiary	3.9	4.3	3.7
Household wealth quintile, %			
Lowest	19.7	19.0	20.1
2nd lowest	26.7	26.0	27.1
Middle	24.3	24.7	24.0
2nd highest	16.5	16.6	16.4
Highest	12.9	13.7	12.4
Multiple partners in past 12 mo, %	3.3	7.2	0.6
Any inconsistent condom use in past 12 mo, %	37.4	29.1	42.9
Knows own HIV status, %	29.3	20.9	34.1

Abbreviations: HIV, human immunodeficiency virus; SD, standard deviation.

^a^ Data represent % of respondents unless otherwise specified.

Table 2 shows HIV incidence by partnership status. We observed 1619 HIV seroconversions in 17 106 individuals over 60 349 person-years follow-up time and the overall HIV incidence was 2.7 new infections per 100 PY (95% confidence interval [CI], 2.6–2.8/100 PY). HIV incidence was 5.6/100 PY (95% CI, 3.5–8.4/100 PY) among individuals with an HIV-infected partner not receiving ART, 1.4/100 PY (.4–3.5/100 PY) among those with an HIV-infected partner receiving ART, and 0.3/100 PY (.2–.5/100 PY) among those with an HIV-uninfected partner. Men with HIV-uninfected partners had a higher HIV incidence than women with HIV-uninfected partners, whereas women with HIV-infected partners had higher HIV incidence than men with HIV-infected partners.

**Table 2.** Human Immunodeficiency Virus Incidence by Partner Status and Sex

Partner Status	Overall			Women			Men		
	HIV Seroconversions, No.	PY	Incidence Rate, Seroconversions/100 PY	HIV Seroconversions, No.	PY	Incidence Rate, Seroconversions/100 PY	HIV Seroconversions, No.	PY	Incidence Rate, Seroconversions/100 PY
HIV uninfected	20	6644	0.3 (.2–.5)	8	3720	0.2 (.1–.4)	12	2923	0.4 (.2–.7)
HIV infected	27	707	3.8 (2.3–5.6)	20	406	4.9 (3.0–7.6)	7	302	2.3 (.9–4.8)
Receiving ART	4	294	1.4 (.4–3.5)	4	179	2.2 (.6–5.7)	0	116	0 (0–3.2)
Not receiving ART	23	413	5.6 (3.5–8.4)	16	227	7.0 (4.0–11.4)	7	186	3.8 (1.5–7.8)
Unknown partner status	197	10 713	1.8 (1.6–2.1)	148	8492	1.7 (1.5–2.0)	49	2221	2.2 (1.6–2.9)
In a noncohabiting relationship	78	2171	3.6 (2.8–4.5)	64	1632	3.9 (3.0–5.0)	14	538	2.6 (1.4–4.4)
Not in a relationship	1297	40 114	3.2 (3.1–3.4)	1003	25 335	4.0 (3.7–4.2)	294	14 779	2.0 (1.8–2.2)
Overall	1619	60 349	2.7 (2.6–2.8)	1243	39 586	3.1 (3.0–3.3)	376	20 763	1.8 (1.6–2.0)

Abbreviations: ART, antiretroviral therapy; HIV, human immunodeficiency virus; PY, person-years.

All partnership categories had significantly elevated HIV incidence compared with individuals in stable partnerships with HIV-uninfected partners (Table 3). Individuals with an HIV-infected stable partner had the greatest elevation in HIV incidence, with incidence >10 times that of individuals with HIV-uninfected stable partners (adjusted hazard ratio, 10.04; 95% CI, 5.51–18.32; Table 3). This result was robust to adjustment for factors related to socioeconomic status and sexual behavior (Table 3). The use of ART by an HIV-infected partner was associated with a 77% reduction in HIV incidence compared with no ART use by an HIV-infected partner (adjusted hazard ratio, 0.23; 95% CI, .07–.80; Table 4 and Figure 1). As expected, this effect was slightly stronger than that estimated by the midpoint imputed Cox proportional hazards model (adjusted hazard ratio, 0.28; 95% CI, .10–.80; Supplementary Table 4), with a slightly wider CI. Our results were robust to assuming no effect of ART during the first 30 days after ART initiation (Supplementary Table 5).

**Table 3.** Association Between Partner Serostatus and Human Immunodeficiency Virus Acquisition

Characteristic	Model 1^a^		Model 2^a^		Model 3^a^	
	AHR (95% CI)	*P* Value	AHR (95% CI)	*P* Value	AHR (95% CI)	*P* Value
HIV-uninfected partner	1.00	…	1.00	…	1.00	…
HIV-infected partner	9.91 (5.48–17.94)	<.001	9.71 (5.36–17.60)	<.001	10.04 (5.51–18.32)	<.001
Unknown partner status	4.26 (2.63–6.89)	<.001	4.26 (2.63–6.90)	<.001	4.36 (2.67–7.13)	<.001
In a noncohabiting relationship	6.61 (3.94–11.06)	<.001	6.63 (3.95–11.10)	<.001	6.26 (3.71–10.58)	<.001
Not in a relationship	5.98 (3.73–9.60)	<.001	6.02 (3.75–9.66)	<.001	6.42 (3.97–10.40)	<.001
Female sex	2.19 (1.94–2.46)	<.001	2.18 (1.93–2.45)	<.001	2.24 (1.98–2.54)	<.001
Age at baseline	1.13 (1.10–1.16)	<.001	1.13 (1.10–1.16)	<.001	1.11 (1.08–1.12)	<.001
Age squared	0.998 (.997 to.998)	<.001	0.998 (.997 to.998)	<.001	0.998 (.997 to.999)	<.001
Educational attainment						
None or primary (0–7 y)	…	…	1.00	…	1.00	…
Secondary (8–12 y)	…	…	0.99 (.86–1.13)	.85	0.93 (0.81–1.07)	.31
Tertiary	…	…	0.94 (.73–1.22)	.66	0.88 (.67–1.14)	.33
Household wealth quintile						
Lowest	…	…	1.00	…	1.00	…
2nd lowest	…	…	1.20 (1.02–1.41)	.03	1.17 (.99–1.37)	.07
Middle	…	…	1.28 (1.09–1.41)	.002	1.28 (1.09–1.50)	.003
2nd highest	…	…	1.24 (1.06–1.46)	.008	1.24 (1.05–1.46)	.01
Highest	…	…	1.06 (.89–1.26)	.54	1.08 (.90–1.28)	.41
Multiple partners in past 12 mo	…	…	…	…	2.25 (1.76–2.88)	<.001
Any inconsistent condom use in past 12 mo	…	…	…	…	1.72 (1.56–1.92)	<.001

Abbreviations: AHR, adjusted hazard ratio; CI, confidence interval; HIV, human immunodeficiency virus.

^a^ The 3 models are described in “Statistical Methods” section.

**Table 4.** Association Between Partner Antiretroviral Therapy Status and Human Immunodeficiency Virus Acquisition

Characteristic	Model 1^a^		Model 2^a^		Model 3^a^	
	AHR (95% CI)	*P* Value	AHR (95% CI)	*P* Value	AHR (95% CI)	*P* Value
HIV-infected partner, not receiving ART	1.00	…	1.00	…	1.00	…
HIV-infected partner, receiving ART	0.23 (.07–.80)	.02	0.23 (.07–.80)	.02	0.23 (.07–.80)	.02
HIV-uninfected partner	0.08 (.05–.15)	<.001	0.09 (.05–.16)	<.001	0.08 (.04–.15)	<.001
Unknown partner status	0.32 (.21–.50)	<.001	0.33 (.21–.51)	<.001	0.32 (0.21–.50)	<.001
In a noncohabiting relationship	0.50 (.31–.80)	.004	0.51 (.32–.82)	.005	0.46 (.29–.73)	.001
Not in a relationship	0.46 (.30–.70)	.0003	0.46 (.30–.70)	.0004	0.47 (.31–.72)	.0005
Female sex (No., %)	2.19 (1.95–2.46)	<.001	2.18 (1.94–2.45)	<.001	2.25 (1.99–2.54)	<.001
Age at baseline	1.13 (1.10–1.16)	<.001	1.13 (1.10–1.16)	<.001	1.11 (1.08–1.14)	<.001
Age squared	0.998 (.997–.998)	<.001	0.998 (.997–.998)	<.001	0.998 (.997–.998)	<.001
Educational attainment						
None or primary (0–7 y)	…	…	1.00	…	1.00	…
Secondary (8–12 y)	…	…	0.99 (.86–1.13)	.84	0.93 (.81–1.07)	.33
Tertiary	…	…	0.94 (.72–1.22)	.62	0.89 (.68–1.15)	.37
Household wealth quintile						
Lowest	…	…	1.00	…	1.00	…
2nd lowest	…	…	1.20 (1.02–1.41)	.03	1.17 (.99–1.38)	.06
Middle	…	…	1.28 (1.09–1.51)	.003	1.28 (1.09–1.51)	.003
2nd highest	…	…	1.24 (1.06–1.46)	.008	1.24 (1.05–1.45)	.01
Highest	…	…	1.06 (.89–1.26)	.53	1.07 (.90–1.27)	.43
Multiple partners in past 12 mo	…	…	…	…	2.26 (1.76–2.89)	<.001
Any inconsistent condom use in past 12 mo	…	…	…	…	1.73 (1.56–1.92)	<.001

Abbreviations: AHR, adjusted hazard ratio; ART, antiretroviral therapy; CI, confidence interval; HIV, human immunodeficiency virus.

^a^ The 3 models are described in “Statistical Methods” section.

## DISCUSSION

In controlled and resource-intensive randomized trials and clinical cohort studies, early initiation of ART dramatically reduces HIV transmission to uninfected partners in serodiscordant relationships [12, 15, 39]. In the landmark HPTN-052 study, immediate ART initiation resulted in a 96% reduction in linked HIV transmission and 89% reduction in overall HIV transmission among serodiscordant couples [12]. In the present study, we estimated the effect of ART in reducing HIV acquisition in serodiscordant couples in a “real-life” population-based setting. We found that ART is highly effective in reducing HIV acquisition in serodiscordant couples in a poor HIV-hyperendemic community, despite severe resource constraints in the public-sector health system in this setting.

In this community, ART is delivered through primary care clinics that are staffed and led exclusively by nurses. Supply chain failures in this setting have frequently led to drug stockouts during the observation period, patients commonly miss scheduled visits to the ART program, and adherence to ART is lower than in more tightly controlled study settings. Despite these imperfections of “real-life” ART delivery, our results demonstrate that ART is highly effective in preventing HIV transmission in serodiscordant couples. Because this study was population-based, including all stable discordant couples in the community, our findings are generalizable beyond those of the previous controlled clinical studies, which enrolled only individuals who, firstly, used healthcare targeting couples and, secondly, had disclosed their HIV status to their partners [12, 20–24, 40].

Our results must be considered in the context of several limitations. First, as with any long-term population-based study, there was some attrition and nonresponse. Selection bias could be introduced if those who tested systematically differed from those who declined testing. Second, we cannot rule out bias due to unmeasured confounding. For example, individuals with greater general engagement with health services may be both more likely to initiate ART and more likely to use condoms. However, the longitudinal nature of this analysis allowed for adjustment of time-varying confounding by a range of sociodemographic and behavioral factors that probably at least partially control for such effects. Importantly, the large effect sizes in this study mean that any bias due to confounding would have to be considerable to nullify these results.

Third, although this study represents a true population cohort, our findings may not be generalizable to all contexts. In rural South Africa, cohabitation and conjugal relationships tend to occur at older ages [35], whereas HIV incidence is highest in younger men and women [14, 29]. Sexual partnership structures similar to those observed in our study population are also found in other poor rural settings in Southern Africa. However, our results may not be generalizable to communities with different partnerships structures, for example, where serodiscordant partnerships are prevalent among younger individuals. Finally, the observed HIV incidence among individuals with HIV-uninfected stable partners was relatively low, and although this may partially reflect overall lower HIV risk behaviors among this population, it may also reflect a selection effect: in this setting of very high HIV prevalence, those who have not already acquired HIV by the time they enter a stable partnership may have lifelong risk behaviors that are qualitatively different from the rest of the population.

In conclusion, ART is a highly effective strategy to prevent HIV transmission in serodiscordant couples, even when it is provided to a population with low levels of HIV status disclosure and through a resource-poor, public-sector healthcare system in a hyperendemic community. Although the HIV prevention effect of ART in this “real-life” setting is large, it falls short of the near-complete elimination of HIV transmission observed in the HPTN-052 study, suggesting that additional prevention interventions will probably be required to eliminate HIV transmission in serodiscordant couples in SSA.

## Supplementary Data

Supplementary materials are available at http://cid.oxfordjournals.org. Consisting of data provided by the author to benefit the reader, the posted materials are not copyedited and are the sole responsibility of the author, so questions or comments should be addressed to the author.

Supplementary Data
