# Supplementary material for: Antiretroviral Therapy to Prevent HIV Acquisition in Serodiscordant Couples in a Hyperendemic Community in Rural South Africa
Source: Clin Infect Dis. 2016 May 20;63(4):548–54. doi: 10.1093/cid/ciw335 (PMC4967606; doi:10.1093/cid/ciw335)
Supplement: Supplementary Data [file supp_63_4_548__index.html]

Antiretroviral Therapy to Prevent HIV Acquisition in Serodiscordant Couples in a Hyperendemic Community in Rural South Africa — Supplementary Data 

# Antiretroviral Therapy to Prevent HIV Acquisition in Serodiscordant Couples in a Hyperendemic Community in Rural South Africa

## Supplementary Data

Supplementary Data

- Supplementary Data - Docx file
