# Supplementary material for: Antiretroviral Therapy to Prevent HIV Acquisition in Serodiscordant Couples in a Hyperendemic Community in Rural South Africa
Source: Clin Infect Dis. 2016 May 20;63(4):548–54. doi: 10.1093/cid/ciw335 (PMC4967606; doi:10.1093/cid/ciw335)
Supplement: Supplementary Data [file supp_ciw335_ciw335supp.docx]

**SUPPLEMENTARY INFORMATION**

**Table S1.** Summary of published longitudinal studies of treatment as prevention for heterosexual serodiscordant couples

| **Author [Year]** | **N** | **Country** | **Time Period** | **Study design** | **Effect estimate** | **Mutually Disclosed?** | **Method for dealing with interval censoring** |
| --- | --- | --- | --- | --- | --- | --- | --- |
| Birungi [2015] | 586 | Uganda | 2009-2011 | Prospective cohort | 1.07 [0.41-2.08]^1^  (unadjusted) | Yes | NR |
| Tang [2015] | 6,548 | China | 2003-2013 | Prospective cohort | 0.65 [0.51-0.83]^2^ | Yes | NR |
| Smith [2015] | 4,916 | China | 2006-2012 | Prospective cohort | 0.52 [0.34-0.82]^3^ | Yes | Midpoint |
| Yang [2015] | 1,258 | China | 2005-2007 | Prospective cohort | 0.02 [0.001-0.18]^4^ | Yes | Cumulative incidence |
| Zhang [2015] | 1,854 | China | 1996-2013 | Retrospective cohort | 0.45 [0.28-0.71]^5^ | Yes | NR |
| He [2013] | 1,101 | China | 2009-2011 | Prospective cohort | 0.30 [0.10-0.86]^6^ | Yes | Midpoint |
| Jean [2014] | 957 | Côte d’Ivoire | 2009-2011 | Randomized open-label trial | 0.10 [0.05 to 0.19]^7^ | No | N/A |
| Biraro [2013] | 259 | Uganda | 1989-2007 | Prospective cohort | 0^8^ | No | Midpoint |
| Jia [2013] | 38,862 | China | 2003-2011 | Retrospective cohort | 0.74 [0.65-0.84]^9^ | Yes | NR |
| Reynolds [2011] | 251 | Uganda | 2004-2009 | Prospective cohort | 0^10^ | No | NR |
| Cohen [2011] | 1,763 | Botswana, Kenya, Malawi, South Africa, Zimbabwe, Brazil, India, Thailand, United States | 2007-2011 | Randomized controlled trial | 0.04 [0.01 to 0.27]^11^ | Yes | NR |
| Wang [2010] | 1,927 | China | 2006-2008 | Retrospective cohort | 1.32 [0.78-2.22]^12^ | Yes | Actual date |
| Donnell [2010] | 3,381 | Botswana, Kenya, Rwanda, South Africa, Tanzania, Uganda, Zambia | 2004-2007 | Prospective cohort | 0.08 [0.00-0.57]^13^ | Yes | Monitoring done every 3 months; any 3-month period in which ART was used considered ART-exposed |
| Del Romero [2010] | 476 | Spain | 1989-2008 | Prospective cohort | 0^14^ | Yes | NR |
| Melo [2008] | 93 | Brazil | 2000-2006 | Retrospective cohort | 0^15^ | Yes | NR |
| Bunnell [2006] | 926 | Uganda | 2003-2004 | Prospective cohort | 98% estimated reduction^16^ | No | N/A |
| Castilla [2005] | 393 | Spain | 1991-2003 | Prospective cohort | 0.14 [0.03-0.66]^17^ | Yes | Cumulative incidence |
| Musicco [1994] | 436 | Italy | 1987-1992 | Prospective cohort | 0.50 [0.10-0.90]^18^ | Yes | Midpoint |

Abbreviations: NR=not reported; N/A=not applicable; ^1^Time-updated univariate Cox proportional hazards model; ^2^Multivariable Cox proportional hazards model adjusted for duration of follow-up, sex, age, education, marital status, occupation, route of HIV infection, baseline CD4 cell count in index patient; ^3^Time-varying marginal structural model adjusted for age, sex, education, disease stage, time period; ^4^Multivariable logistic regression model adjusted for age, viral load in index partner, and condom use in the past 6 months; ^5^Multivariable Cox proportional hazards model adjusted for sex, index partner CD4 count, frequency of condom use in the past 3 months; ^6^Multivariable Cox proportional hazards model adjusted for age, sex, education, HSV-2 serostatus in each partner, and frequency of sex in the past 12 months; ^7^Model based on estimated per-coital-act probability of transmission in the early ART initiation group compared to the delayed ART initiation group; ^8^No HIV seroconversions seen in 29 couples in which the index partner had initiated ART; ^9^Multivariable Cox proportional hazards model adjusted for duration of follow-up, sex, age, education, marital status, occupation, route of HIV infection, and baseline CD4 count in the index partner; ^10^No HIV seroconversions among 32 HIV-infected index partners who received ART; ^11^Cox proportional hazards model; ^12^Univariate Cox proportional hazards model; ^13^Exact Poisson model adjusted for time on study and CD4 cell count; ^14^No HIV seroconversions seen in 144 couples; No HIV seroconversions seen in 41 couples; ^16^Estimated reduction in HIV transmission 6 months after initiating ART, based on number of partners, condom use, partner HIV status, frequency of sexual behavior, and viral load; ^17^Multivariable logistic regression model assessing HIV prevalence in the late HAART era (1999-2003) compared to pre-HAART (1991-1995) adjusted for condom use in the past 6 months, CD4 count of the index case, AIDS-defining diseases in the index case, sex, whether the relationship formed before or after HIV infection diagnosis, time since beginning of the relationship, any sexually transmitted infection in the index case, and previous pregnancies; ^18^Multivariable Cox proportional hazards model adjusting for condom use, peno-anal and peno-oral sex, indicators of the index partner’s disease progression

**Table S2.** Demographic and behavioral characteristics by baseline relationship status

|  | Co-habiting Relationship,  Partner Status Known | Co-habiting Relationship, Partner Status Unknown | Non-cohabiting Relationship | Not in Relationship |
| --- | --- | --- | --- | --- |
| Mean age at baseline (standard deviation) | 50.0 (14.6) | 46.0 (15.1) | 32.9 (15.3) | 28.0 (18.9) |
| Educational attainment  None or primary (0-7 years)  Secondary (8-12 year)  Tertiary | 1,255/1,839 (68.2%)  434/1,839 (23.6%)  150/1,839 (8.2%) | 1,286/2,045 (62.9%)  628/2,045 (30.7%)  131/2,045 (6.4%) | 144/444 (32.4%)  261/444 (58.8%)  39/444 (8.8%) | 3,717/11,356 (32.7%)  7,345/11,356 (64.7%)  294/11,356 (2.6%) |
| Household wealth quintile  Lowest  2^nd^ lowest  Middle  2^nd^ highest  Highest | 406/1,925 (21.1%)  530/1,925 (27.5%)  441/1,925 (22.9%)  295/1,925 (15.3%0  253/1,925 (13.1%) | 359/1,745 (20.6%)  498/1,745 (28.5%)  413/1,745 (23.7%)  262/1,745 (15.0%)  213/1,745 (12.2%) | 88/415 (21.2%)  116/415 (28.0%)  101/415 (24.3%)  61/415 (14.7%)  49/415 (11.8%) | 1,956 /10,193 (19.2%)  2,669/10,193 (26.2%)  2,507/10,193 (24.6%)  1,733/10,193 (17.0%)  1,328/10,193 (13.0%) |
| Multiple partners, past 12 months | 43/1,424 (3.0%) | 29/1,763 (1.6%) | 20/478 (4.2%) | 372/10,628 (3.5%) |
| Any inconsistent condom use, past 12 months | 951/1,424 (66.8%) | 1,189/1,762 (67.5%) | 311/479 (64.9%) | 2,891/10,630 (27.2%) |

Sensitivity analysis using midpoint imputation

This analysis using a traditional midpoint imputation for determination of HIV seroconversion date with a Cox proportional hazards model

**Table S3.** Association between partner serostatus and HIV acquisition

|  | **MODEL 1^1^** | | **MODEL 2^2^** | | **MODEL 3^3^** | |
| --- | --- | --- | --- | --- | --- | --- |
|  | Hazards Ratio  (95% CI) | *P*-value | Hazards Ratio  (95% CI) | *P*-value | Hazards Ratio  (95% CI) | *P*-value |
| **Negative partner**  **Positive partner**  Unknown partner status  In relationship, non-conjugal  Not in relationship | 1.00  **8.34 (4.67 to 14.89**  4.05 (2.55 to 6.43)  5.38 (3.27 to 8.86)  5.97 (3.79 to 9.38) | **<0.001**  <0.001  <0.001  <0.001 | 1.00  **8.32 (4.66 to 14.87)**  3.75 (2.36 to 5.96)  5.18 (3.13 to 8.55)  5.67 (3.61 to 8.93) | **<0.001**  <0.001  <0.001  <0.001 | 1.00  **8.70 (4.83 to 15.67)**  3.88 (2.42 to 6.25)  5.11 (3.07 to 8.53)  6.08 (3.82 to 9.68) | **<0.001**  <0.001  <0.001  <0.001 |

^1^Adjusted for sex, age, and visit year; ^2^Adjusted for sex, age, visit year, educational attainment, and household wealth; ^3^Adjusted for sex, age, visit year, educational attainment, household wealth, and sexual behaviors

**Table S4.** Association between partner ART status and HIV acquisition

|  | **MODEL 1^1^** | | **MODEL 2^2^** | | **MODEL 3^3^** | |
| --- | --- | --- | --- | --- | --- | --- |
|  | Hazards Ratio  (95% CI) | *P*-value | Hazards Ratio  (95% CI) | *P*-value | Hazards Ratio  (95% CI) | *P*-value |
| **Positive partner, not on ART**  **Positive partner, on ART**  Negative partner  Unknown partner status  In relationship, non-conjugal  Not in relationship | 1.00  **0.28 (0.10 to 0.81)**  0.09 (0.05 to 0.16)  0.35 (0.23 to 0.54)  0.47 (0.29 to 0.75)  0.52 (0.34 to 0.79) | **0.02**  <0.001  <0.001  0.002  0.002 | 1.00  **0.28 (0.10 to 0.82)**  0.09 (0.05 to 0.16)  0.33 (0.21 to 0.51)  0.45 (0.28 to 0.73)  0.50 (0.32 to 0.76) | **0.02**  <0.001  <0.001  0.001  0.001 | 1.00  **0.28 (0.10 to 0.80)**  0.09 (0.05 to 0.15)  0.32 (0.21 to 0.50)  0.42 (0.26 to 0.68)  0.50 (0.33 to 0.77) | **0.02**  <0.001  <0.001  <0.001  0.001 |

^1^Adjusted for sex, age, and visit year; ^2^Adjusted for sex, age, visit year, educational attainment, and household wealth; ^3^Adjusted for sex, age, visit year, educational attainment, household wealth, and sexual behaviors

Primary analysis using “wash-in” period of 30 days for ART initiation

This analysis uses an interval censored parametric survival model and assumes a 30 day “wash in” period for ART; that is, that ART does not have an effect until 30 days after initiation.

**Table S5.** Association between partner ART status and HIV acquisition

|  | **MODEL 1^1^** | | **MODEL 2^2^** | | **MODEL 3^3^** | |
| --- | --- | --- | --- | --- | --- | --- |
|  | Hazards Ratio  (95% CI) | *P*-value | Hazards Ratio  (95% CI) | *P*-value | Hazards Ratio  (95% CI) | *P*-value |
| **Positive partner, not on ART**  **Positive partner, on ART**  Negative partner  Unknown partner status  In relationship, non-conjugal  Not in relationship | 1.00  **0.23 (0.06 to 0.79)**  0.09 (0.05 to 0.16)  0.32 (0.21 to 0.50)  0.50 (0.31 to 0.80)  0.46 (0.30 to 0.70) | **0.02**  <0.001  <0.001  0.004  0.0003 | 1.00  **0.22 (0.06 to 0.79)**  0.09 (0.05 to 0.16)  0.33 (0.21 to 0.51)  0.51 (0.32 to 0.82)  0.47 (0.30 to 0.71) | **0.02**  <0.001  <0.001  0.006  0.0004 | 1.00  **0.22 (0.06 to 0.79)**  0.08 (0.04 to 0.15)  0.32 (0.21 to 0.50)  0.46 (0.29 to 0.74)  0.48 (0.31 to 0.73) | **0.02**  <0.001  <0.001  0.001  0.0006 |

^1^Adjusted for sex, age, and visit year; ^2^Adjusted for sex, age, visit year, educational attainment, and household wealth; ^3^Adjusted for sex, age, visit year, educational attainment, household wealth, and sexual behaviors

**Missing Data**

There was some missing data for some covariates included in multivariable models. For educational attainment, data was available for 15,684/17,016 (92.2%) of participants. Household wealth was available for 14,278/17,016 (83.9%) participants, and sexual behaviors were available for 14,293/17,016 (84.0%) participants. Results reported in the paper are from complete case analysis of multivariable models.
